# Teaching strategies for coping with stress – the perceptions of medical students

**DOI:** 10.1186/1472-6920-13-50

**Published:** 2013-04-08

**Authors:** Maria Amelia Dias Pereira, Maria Alves Barbosa

**Affiliations:** 1 Departamento de Saúde Mental e Medicina Legal da Faculdade de Medicina da Universidade Federal de Goiás - Primeira Avenida, Goiânia, Goiás, Brazil; 2 Faculdade de Enfermagem da Universida de Federal de Goiás, Faculdade de Enfermagem– Rua 227, quadra 68, s/n, Setor Leste Universitário, Goiânia, Goiás, CEP: 74605 080, Brazil

**Keywords:** Stress, Coping strategies, Medical under graduate education, Teaching strategies

## Abstract

**Background:**

The undergraduate medical course is a period full of stressors, which may contribute to the high prevalence of mental disorders among students and a decrease in life’s quality. Research shows that interventions during an undergraduate course can reduce stress levels. The aim of this paper is to evaluate the Strategies for Coping with Professional Stress class offered to medical students of the Federal University of Goiás, at Goiânia, Goiás, in Brazil.

**Methods:**

Qualitative research, developed with medical students in an elective class addressing strategies for coping with stress after a focal group (composed of nine of the 33 students taking this course) identified stress factors in the medical course and the coping strategies that these students use. Analysis of the results of the class evaluation questionnaire filled out by the students on the last day of class.

**Results:**

Stress factors identified by students in the focus group: lack of time, excessive class content, tests, demanding too much of themselves, overload of extracurricular activities, competitiveness among students and family problems. Coping strategies mentioned in the focus group: respecting one’s limits, setting priorities, avoiding comparisons, leisure activities (movies, literature, sports, meeting with friends and family). Results of the questionnaires: class content that was considered most important: quality of life, strategies for coping with stress, stress factors, assertiveness, community therapy, relaxation, cognitive restructuring, career choice, breathing, social networking, taking care of the caregiver, music therapy and narcissism. Most popular methodologies: relaxation practice, drawing words and discussion them in a group, community therapy, music therapy, simulated jury, short texts and discussion. Meaning of the class: asking questions and reinforcing already known strategies (22.6%), moment of reflection and self-assessment (19.4%), new interest and a worthwhile experience (19.4%), improvement in quality of life (16.1%), expression’s opportunity (9.7%), other (6.4%).

**Conclusion:**

The stressors perceived by the medical students are intense and diverse, and the coping strategies used by them are wide-ranging. Most students felt that the class was a worthwhile learning experience, incorporated new practices for improving quality of life and recognized the importance of sharing and reflecting on one’s stressors and life choices.

## Background

An undergraduate course in medicine is a period that creates stress and anxiety in the student since the course is considered difficult, demanding and highly competitive, both for admission and over the course’s 6-year duration [1]. Medicine is characterized by many demands on oneself, social expectations and excessive responsibility. Studies show that students of medicine, nursing, nutrition and other majors get sick or suffer a reduction in quality of life during the course [2]. There is a high prevalence of depression, anxiety and stress [3]. Various factors lead to this situation, especially an excess of study (in terms of both course content and hours), lack of time for leisure activities or relaxation, the difficulty in acquiring new content, students’ demanding too much of themselves and contact with death and suffering [4]. In are view of Brazilian studies the author concluded that the symptoms of anxietyand depression are prevalent during the training of physicians and influence their way of dealing with the profession, their health and their future patients [5-7].

There are few studies showing the effectiveness of support interventions on medical students and residents. In a study with two years of follow up. A three-month study of the impact of group intervention on third-year medical students showed a reduction in the level of stress [8].

Shapiro et al. (2000) found over 600 articles from 1966 to 1999 discussing the importance of stress in medical education but only 24 studies reporting intervention programs and support for students and/or medical residents [9]. In general, the functioning of these programs provided: 1 - An improvement in students’ immunity; 2-Decreased depression and anxiety; 3-increased spirituality and empathy; 4 - Better knowledge of alternative therapies for future reference; 5-Better knowledge of stress; 6-Increased use of positive coping skills; 7-Ability to resolve role conflicts. The primary task of medical schools is to provide students a space where they can reflect on their feelings and emotions, where their vulnerabilities, limitations and conditions can be seen, accepted, cared for and treated when necessary [10].

The Faculty of Medicine, Federal University of Goiás (FM/UFG)is a public Brazilian medical school, founded in 1960 and with a traditional teaching methodology. At the moment,a project is underway to gradually transform the curriculum. The object is to provide professionals with a generalistic, humanistic, critical and reflexive education. The course of medicine is full-time and lasts for six years (the last two years consist of internship training) and the total class load is 9.806 hours. Four hundred and sixty hours are spent on elective subjects [11].

The FM/UFG, in 2011, offered a one-semester elective class whose purpose was to discuss strategies of coping with professional stress. The class syllabus developed as the class unfolded, taking into account the interests and needs ofthe students who attended. This article shows the main stress factors identified by students and the strategies used bythem in dealing with everyday stress. It also presents the content covered and the methodologies used in the class. Finally, this article presents and discusses the results of the class evaluation questionnaire filled out by the students on the last day of class.

## Methods

This is a descriptive and exploratory study. Qualitative data were obtained through focus groups and quantitative data were derived from an instrument containing questions evaluating the elective course about strategies for coping with professional stress offered to medical students.

The research was conducted at the Faculty of Medicine, Federal University of Goiás (UFG) in the city of Goiânia, state of Goiás, center west region of Brazil.

Thirty-three students attended the “Strategies for coping with professional stress” class, of whom nine spontaneously agreed to participate in a focus group to identifystress factors in medical school and the strategies used to address them. The study protocol was approved bythe HC/UFG Research Ethics Committee under Protocol No. 027/2011.

The class, given by the researcher and a psychologist, was offered to medical students as an elective and totaled 32 class hours, with one class per week. Class content was developed taking into account both data from the literature and complaints from students in the focus group at the beginning of the semester.

### A focus group

Is a technique that makes it possible to discover participant’s perceptions, beliefs, values, attitudes and social representations in regard to a specific topic (in our case, stressors in medical school and the strategies used to cope with them) [12]. In this research, focus groups were used to better understand the specific context of the students for whom the class was intended [13].

The focus group was conducted early in the first half of 2011, with the participation of nine students enrolled in the “Strategies for coping with professional stress” class. This was an intentional sample, since the invitation was extended to all the participants on the first day of class. The researcher was the coordinator and the session was recorded and later transcribed in full. A Free and Informed Consent Form (FICF) was read and signed.

The guiding questions were:

What are the stress factors perceived during the medicine course? What are the strategies used to cope with them?

### Mixed questionnaire

Some multiple choice questions, some questions with Likert- type responses and some open questions was self-completed by the students. This questionnaire was created and administered by the researcher on the last day of class, after the FICF was read and signed. Questions concerned: (1) evaluations of the class, (2) participation of each student, (3) opinion of teaching methods and class contents and (4) the meaning of the class in personal life. The questionnaires were administered to all the students with the objective of learning about the experiences they had in the class.

## Results

### Focus group (n = 9)

In the focus group students cited the major stressors that they perceive in the undergraduate medicine course and gave the strategies they use to improve their quality of life. List 1 shows stressors identified by the students in the focus group and List 2 shows coping strategies identified by them.

List 1 Stressors identified by the students in the focus group:

•Tests for different subjects scheduled in the same period;

•Lack of time to study the material to be tested;

•Studying into the night;

•Missing classes;

•The amount of material covered on tests;

•Test subject matter goes beyond what was covered in the classroom;

•The amount of detail required by the teachers;

•Dealing with new forms of assessment such as the OSCE, for example;

•The large amount of extra-curricular activities carried out by students;

•Daily activities unrelated to school (paying bills, cleaning house, etc.);

•Teachers’ lack of time;

•Feelings of guilt because of giving more priority to personal life than to studies;

•Heavy demand on students to study;

•Concern about trying to learn all the content;

•Difficulty in memorizing the content presented;

•Studying material that students consider unnecessary for their professional qualifications;

•Competitiveness among students;

•Waking up very early to go to school;

•Weekday 4th year students weekday on-duty times;

•Family problems;

List 2 Coping strategies used by students in the focus group:

•Skipping classes to perform activities that give pleasure (sports, etc.);

•Identifying with models of physicians who prioritize their own quality of life;

•Study the minimum needed to pass subjects;

•Respect their own physical limits, avoiding spending many hours without sleeping;

•Avoiding comparing grades with other students;

•Going to the movies on weekends;

•Going for walks;

•Getting together with family and friends;

•Cooking;

•Eating well;

•Reading of literary non-medical works;

•Listening to music;

•Dancing;

•Watching football games on television;

•Playing poker;

•Going out to dinner.

Another product of the research was the class itself, constructed with the help of students who participated in the focus groups and as the class progressed, with the joint participation of students and teachers.

In Lists 3 and 4 the content and methods used in the Professional Coping Strategies class offered to medical students in the first half of 2011 are presented.

List 3 Content covered in the “Strategies for coping with professional stress” class:

•Contents:

•Presentation of the students and the syllabus

•Thinking about the concept of quality of life

•Stress factors and what they do to preserve health

•The concept of stress

•Breathing

•Relaxation

•Resilience

•Assertiveness

•Career choice

•Narcissism

•Cognitive restructuring

•Social Networks

•Reevaluation and use of the class

•Communication and self-expression

•Community therapy circles

•Final evaluation and party

•Response to the questionnaires and participation in PROJETO VERAS*

•*PROJECT VERAS (Student's life and resident of the health area): Research project coordinated by the medicine Faculty of Usp (University of São Paulo), with the participation of several medicine faculties in Brazil, including UFG, in process.

Lists 4 Teaching strategies used in the “Strategies for coping with professional stress” class:

•Teaching strategies:

•Self-completion of the stress questionnaire (Lipp)

•Groups of 3 or 4 students reading a text on stress

•Discussion group

•Slideshow on the stress concept

•Teaching the breathing technique

•Writing down the things they do and like, things that they do and don’t like and things that they do not do and would like to do – *“curtograma”*

•Training, progressive relaxation technique, lying on towels on the floor

•Brainstorming on the concept of resilience, what makes things better and what makes things worse

•Slides on the concept of resilience

•Self-completed behavior test, Are you assertive or not?, the concept of assertiveness (classroom dialogue)

•Small group dramatizations of different communication situations. Video

•Simulated jury with an imaginary story, students were divided into three groups for choice of different career paths and discussion

•Texts for homework

•Myth of Narcissus, the psychoanalytic theory of narcissism, classroom dialogue and video

•Breakdown into subgroups for in-class study of the phases of automatic thinking with short texts and presentation in a circle

•Slides on improving standards of automatic thinking

•Mapping of each student’s social network through imagining a possible problem

•Theory of social support

•Music to sensitize reflection, stimulate self-perception

•Circle discussion with a former student who is already in the labor market

•A visiting professor used the specific technique of music therapy. Topic: sound expression (vocal)

•A visiting professor used the community therapy technique. The topic, fear of loss of a loved one, was chosen democratically by the students

•In a circle, students were sensitized to observe their feelings

•Students drew folded slips with a word on them out of a hat and told what that word meant in their lives

•Administration of two self-completed questionnaires, evaluation of the class and its impact on the student’s life; students entered the Projeto Veras site as volunteers after reading the FICF and giving their consent.

### Results of the questionnaires (n = 31)

**Figure 1:**
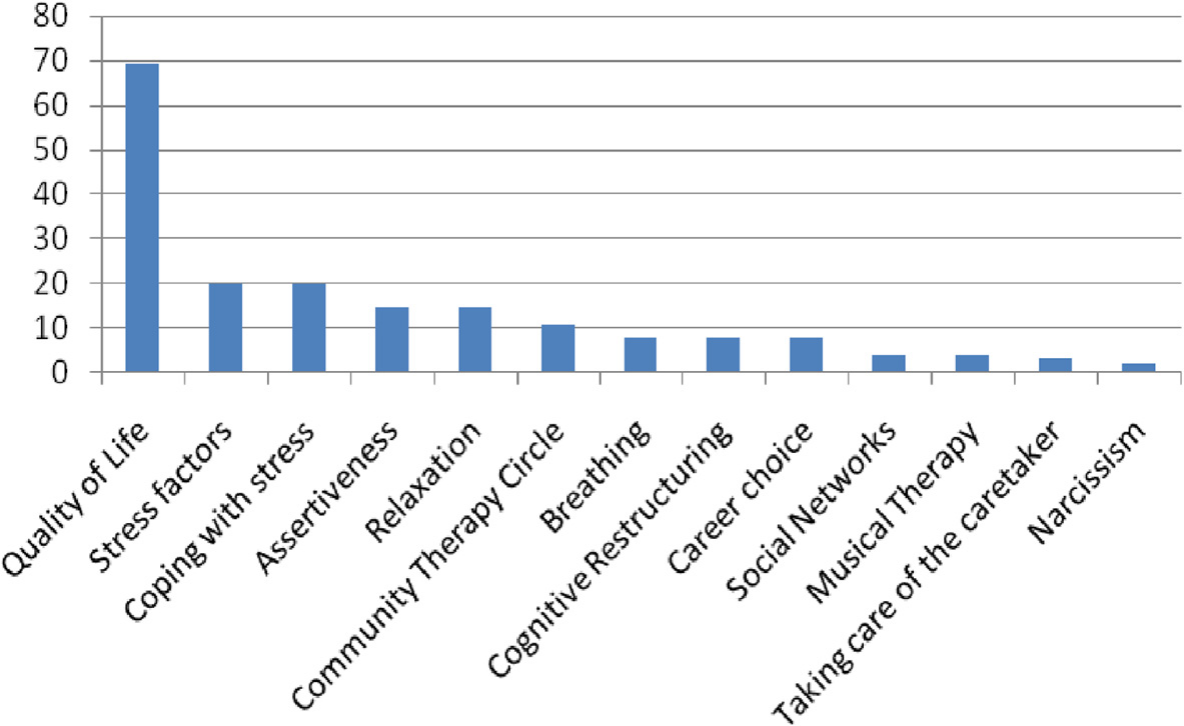
Contents that students liked most (n = 31).

More than half of students (58.1%) were women, 35.5% were enrolled in the third year and the others in the 4th year, ages ranged from 19–26 years. The contents of classes that students liked most are shown in Figure 1.

**Figure 2:**
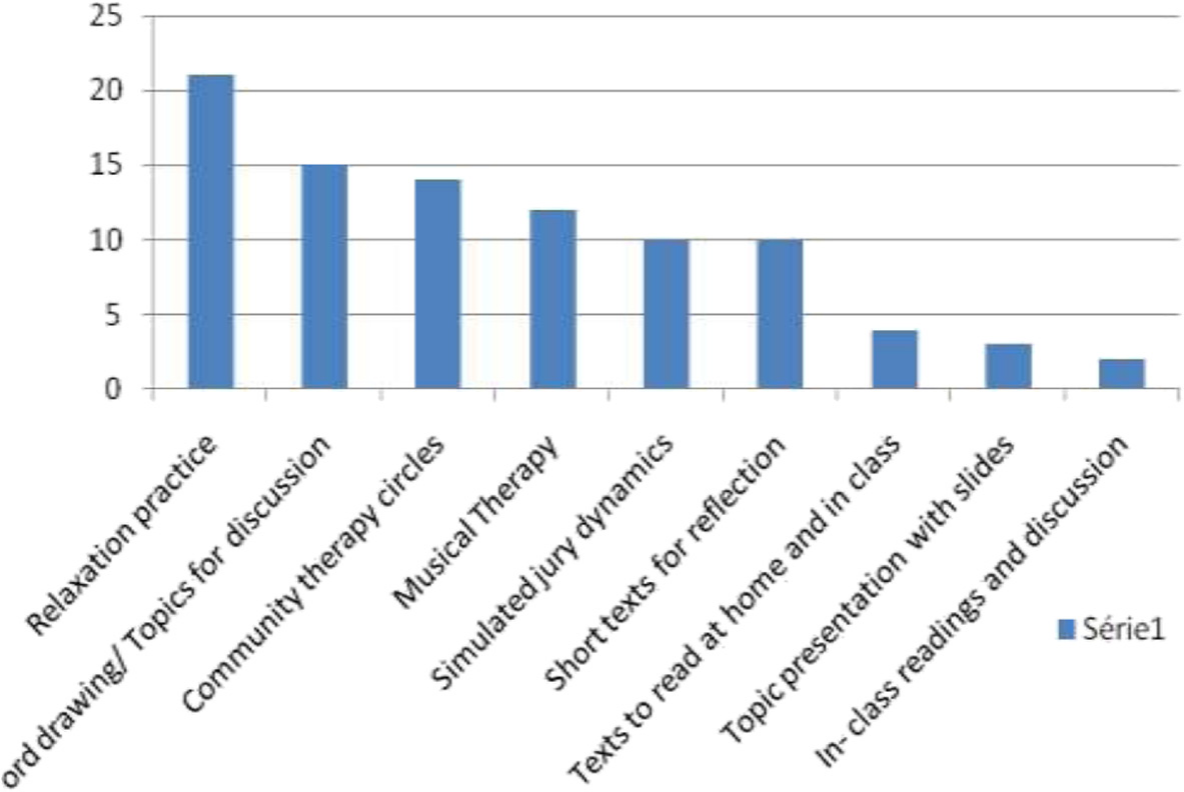
Methods the students liked most (N = 31).

Twenty-five point 8 percent of students would exclude some topic or other, 50% of them would exclude music therapy, 25% would get rid of community therapy and 25% did not specify what they would exclude Figure 2 shows didactic methods used in discipline that students liked most.

Fifty eight percent of the students responded that some methods bothered them, half of these complained of the traditional methods like slides and texts to be read at home and the other half were split between music therapy and the community therapy circle.

On the level of their own participation, students rated their own participation as: good (67.8%), excellent (29%) and weak (3.2%).

The open question of what the class meant for students was answered by 29 and the responses were grouped as follows:

Class cleared up questions, strengthened strategies that I already knew: 22.6%

It was a moment of reflection and self-evaluation: 19.4%

A new interest, a worthwhile experience: 19.4%

Improvement in quality of life: 16.1%

Opportunity for expression: 9.7%

Relaxation: 3.2%

**Figure 3:**
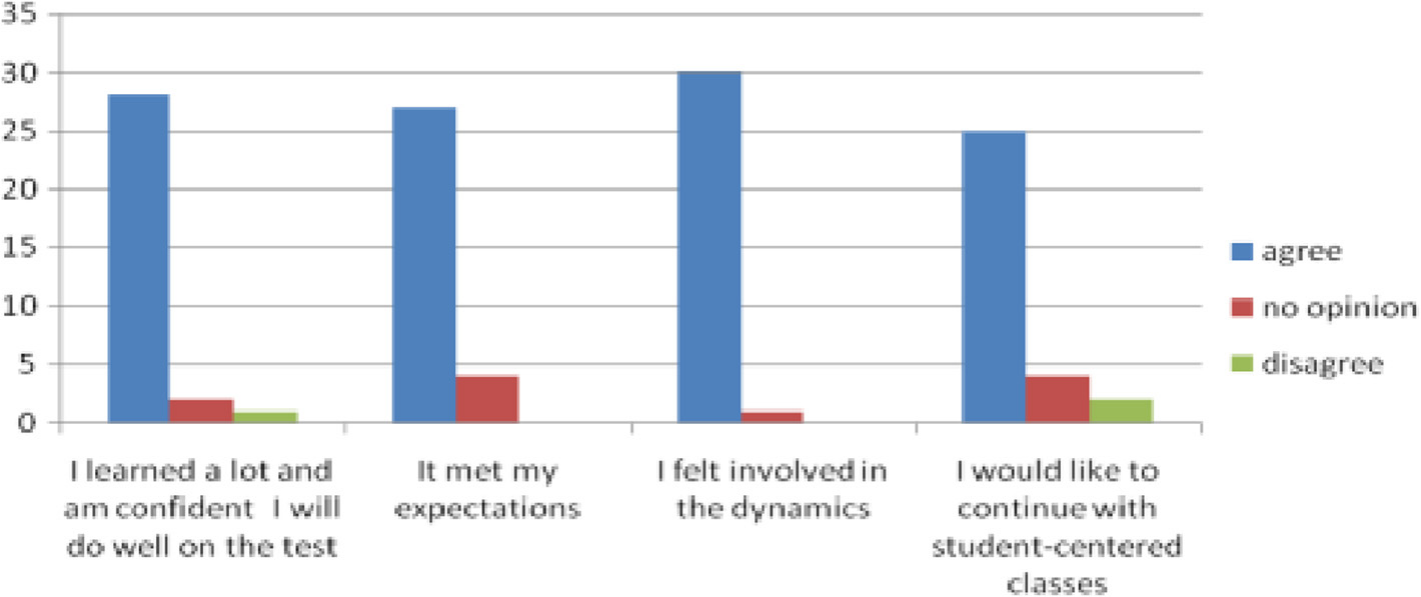
Opinion of students about their learning in the class, their expectations and involvement in the classes and their views on student-centered classes.

The class fulfilled elective requirements: 3.2%. In Figure 3 the graph shows the students' opinions about their learning in the class, their expectations and involvement in the classes and their views on student-centered classes. 

The total class hours were considered ideal by 64.5% of the students and 25.8% of the students thought the number could be higher.

## Discussion

The stressors mentioned by students in the focus group coincide with those found in the literature such as competition, knowledge overload, difficulty in budgeting time among a large number of activities and too little time for leisure activities, individualism, responsibility and social expectations of the doctor’s role [14]. Federal University of Goiás medical students clearly identified their difficulties:

Participant 1: “*What’s missing in medical school is coordination among subjects… often all the tests fall on the same week.*”

Participant 2: *“We don’t have much space for our personal life… we kind of live for the course 24 hours a day. For example… you get an e-mail with a clinical case at 11 pm and you have to study it for the next day at 7 in the morning.”*

Participant 3: *“One of the things that I think most stress students here is what I perceive as the tremendous competitiveness among medical students.”*

Shah et al. (2010) found that among medical students in Pakistan, the most common sources of stress were related to psychosocial and academic concerns, high parental expectations, frequency of examinations, the vastness of the academic curriculum, sleeping difficulties, concern for the future and loneliness [15].

The strategies that the group identified for coping with stress were quite diverse, covering almost all categories found in the research done with this specific population. Examples of what students said:

Participant 1: “..*and I try to see what my limit is, because I think that it is very important for everybody to respect their limits. I have a limit and there comes a time when I can’t study anymore, so I stop, you know, because it doesn’t do any good.”*

Participant 2: *“…one thing I didn’t mention…one thing for me is a priority and that is reading…above and beyond medicine.”*

In a qualitative study at the UFSC Faculty of Medicine, Zonta et al. (2006) identified appreciation of interpersonal relationships and everyday life phenomena, balance between study and leisure, time management, health care, food and sleep, physical activity, religiosity, working on one's own personality to deal with adverse situations and seeking psychological assistance [16].

The topics chosen for the class and the teaching strategies used were consistent with the National Curriculum Guidelines [17], which state that *“the course in medicine should use methodologies that emphasize active student participation in constructing knowledge and integrating content and should stimulate the interaction between teaching, research and extension/service; and the student should look after his/her own physical and mental health and seek well-being as a citizen and as a doctor.” *The dialectical methodology used in this class favors the development of mental operations, providing the student with feelings or moods loaded with personal experience and renewal [18]. In this study, the great majority of students felt involved with the in-class dynamics and reported that they learned a lot and that they would like to continue having learner-centered classes.

The students were quite receptive to this approach, which met the expectations of some and surpassed those of others. On the first day of class several students said they had only chosen this classto meet elective requirements and because the class time was convenient, but on the final questionnaire only one student responded that the class had that meaning for him. Most considered it a worthwhile experience and that it had brought benefits and reflections, with positive effects on their quality of life.

Examples of comments from students:

Participant 1:*“I learned some techniques that were really helpful in coping with daily stress. Learning to be more assertive was particularly important. This had been a real problem for me before and really improved as a result of the elective class.”*

Participant 2*: “I added physical activities, the avoidance of automatic thinking, respect for my desires and limits and spending more time with friends and family to my life.”*

Participant 3: “This class brought up some important points in the area of self-perception and reflection which was great because they helped me revisit some problem areas I had. I have attempted to incorporate breathing practice into my daily life.”

Participant 4: *“The strategies help in a number of ways. But I still think that changes only happen with a deeper and more individual approach. But in the class the strategies help people who don’t think about their behavior to think more and also to relax.”*

It is interesting to note that while one quarter of the students did not like the therapy wheel and music therapy, 14 students included the therapy wheel and 12 included music therapy among their favorite methods. This suggests that it is impossible to develop a curriculum that appeals to 100% of students. We can also speculate that techniques that lead to a greater mobilization of feelings and emotions can be more threatening than others to some people, leading to a higher rejection rate for these techniques, and we need to consider whether it is the role of the educator to make these interventions in the classroom.

Research limitation: the students in the study were third- and fourth-year medical students only and the results cannot be generalized to all medical students.

## Conclusion

The stressors perceived by medical students are intense and diverse, and the coping strategies used by them are wide-ranging.

The “Strategies for coping with professional stress” class proved to be a suitable tool for teaching and learning about the students’ role in taking care of their own health.

For most students the class had a positive meaning. They considered it a worthwhile learning experience, were able to incorporate new practices to improve their quality of life, reinforced practices they already knew, and in particular recognized the importance of having a space and time for sharing with their classmates about the stressors of academic life and reflecting on their attitudes, choices and quality of life.

## Competing interests

The authors declare that they have no competing interests.

## Authors’ contributions

MADP developed and carried out the research Project. She was the coordinator of the focus group, taught the “Strategies for coping with professional stress” class together with psychologist Jussara Miranda, and administered the evaluation questionnaires. MAB was the advisor on the project and collaborated in the writing of the article. Both authors read and approved the final manuscript.

## Pre-publication history

The pre-publication history for this paper can be accessed here:

http://www.biomedcentral.com/1472-6920/13/50/prepub

## References

[B1] Fiorotti KP Rossoni RR Borges LH Miranda AE Transtornos mentais comuns entre os estudantes do curso de medicina: prevalência e fatores associados J Bras Psiquiatr 2010 59 17 23 10.1590/S0047-20852010000100003

[B2] JdM C Rocha FL Prevalência de depressão entre estudantes universitários J Bras Psiquiatr 2006 55 264 267 10.1590/S0047-20852006000400001

[B3] Dyrbye LN Thomas MR Shanafelt TD Systematic review of depression, anxiety, and other indicators of psychological distress among U.S. and Canadian medical students Acad Med 2006 81 4 354 373 10.1097/00001888-200604000-00009 16565188

[B4] De Armond MM A quality assurance program for a Mental Health Service J Am Coll Health Assoc 1981 30 3 139 140 10.1080/01644300.1981.10393057 7328240

[B5] Amaral GF Gomide LMP Batista MP Píccolo PP Teles TBG Oliveira PM Pereira MAD Sintomas depressivos em acadêmicos de medicina da Universidade Federal de Goiás: um estudo de prevalência Revista de Psiquiatria do Rio Grande do Sul 2008 30 124 130

[B6] Baldassin S Ansiedade e Depressão no Estudante de Medicina: Revisão de Estudos Brasileiros Cadernos ABEM 2010 6 6 19 26

[B7] Baldassin S Alves TC de Andrade AG de Nogueira Martins LA The characteristics of depressive symptoms in medical students during medical education and training: a cross-sectional study BMC Med Educ 2008 8 60 10.1186/1472-6920-8-60 19077227 PMC2621219

[B8] Holm M Tyssen R Stordal KI Haver B Self-development groups reduce medical school stress: a controlled intervention study BMC Med Educ 2010 10 23 10.1186/1472-6920-10-23 20233434 PMC2847570

[B9] Shapiro SL Shapiro DE Schwartz GER Stress management in medical education: a review of the literature Acad Med 2000 75 7 748 10.1097/00001888-200007000-00023 10926029

[B10] Millan LR Arruda PCV Assistência psicológica ao estudante de medicina: 21 anos de experiência Rev Assoc Med Bras 2008 54 90 94 10.1590/S0104-42302008000100027 18392493

[B11] UniversidadeFederal deGoiás-Faculdadede Medicina: Projeto Politico Pedagógico: http://www.medicina.ufg.br/uploads/148/original_projetopedagogico.pdf

[B12] Iervolino SA Pelicioni MCF A utilização do grupo focal como metodologia qualitativa na promoção da saúde Rev Esc Enf USP 2001 35 n.2 15 21 10.1590/s0080-62342001000200004 12049046

[B13] Westphal MF Faria MM Grupos focais: experiências precursoras em programas educativos em saúde no Brasil Boletin de la Oficina Sanitaria Panamericana (OSP) 1996 120 6 472 482 8754662

[B14] Benvegnú LA Deitos F Copette FR Problemas psiquiátricos menores em estudantes de Medicina da Universidade Federal de Santa Maria, RS, Brasil Rev Psiquiatr Rio Gd Sul 1996 18 3 229 233

[B15] Shah M Hasan S Malik S Sreeramareddy C Perceived stress, sources and severity of stress among medical undergraduates in a Pakistani medical school BMC Med Educ 2010 10 1 2 10.1186/1472-6920-10-2 20078853 PMC2820489

[B16] Zonta R Robles ACC Grosseman S Estratégias de enfrentamento do estresse desenvolvidas por estudantes de medicina da Universidade Federal de Santa Catarina Revista Brasileira de Educação Médica 2006 30 3 147 153

[B17] Diretrizes Curriculares - Cursos de Graduação: http://portal.mec.gov.br/index.php?option=com_content&view=article&id=12991

[B18] Anastasiou LGC Alves LP Processos de ensinagem na universidade: pressupostos para as estratégias de trabalho em aula 2004 Joinville, SC: Universille

